# Sulforaphane Against the Metabolic Consequences of a High-Glycemic-Index Diet: Protective and Therapeutic Mechanisms Associated with Obesity and Insulin Resistance

**DOI:** 10.3390/nu18040574

**Published:** 2026-02-09

**Authors:** Mihrican Çubuk, Aylin Açıkgöz Pınar, Bahadır Süleyman, Necip Gökhan Taş

**Affiliations:** 1 Department of Nutrition and Dietetics, Hacettepe University, 06100 Ankara, Turkey; aylinn@hacettepe.edu.tr; 2 Department of Nutrition and Dietetics, Erzincan Binali Yıldırım University, 24100 Erzincan, Turkey; 3 Department of Medical Pharmacology, Erzincan Binali Yıldırım University, 24100 Erzincan, Turkey; bsuleyman@erzincan.edu.tr; 4 Department of Medical Microbiology, Erzincan Binali Yıldırım University, 24100 Erzincan, Turkey; necip.tas@erzincan.edu.tr

**Keywords:** sulforaphane, functional food, bioactive compounds, glycemic index, glycemic control, obesity, insulin resistance, dyslipidemia, molecular mechanisms

## Abstract

**Objective:** This study investigated the effects of different doses and timing of sulforaphane (SFN) supplementation on reducing obesity induced by a high-glycemic-index diet (HGID) and on correcting poor glycemic control and dyslipidemia in C57BL/6 mice. **Method:** For 15 weeks, mice were administered a control diet (control), HGID, HGID + oral 5 mg/kg/day SFN (HGID + LSFN), or HGID + 20 mg/kg/day SFN (HGID + HSFN), and following 15 weeks of HGID, mice were treated with 5 mg/kg/day SFN (PO-HGID + LSFN) or 20 mg/kg/day SFN (PO-HGID + HSFN) for 5 weeks. **Results:** SFN reduced body weight gain and serum glucose. The lowest levels of HbA1c were observed in the control and HGID + LSFN groups. Mice in the HGID group exhibited impaired glucose clearance and were less sensitive to insulin compared to the control. A remarkable improvement in glucose and insulin tolerance was observed in both PO-HGID + SFN and HGID + SFN groups. Lipid profile parameters and serum insulin levels were found to be lower in the control and HGID + SFN groups compared to the HGID group. SFN increased serum adiponectin levels when administered concurrently with HGID. IRS1 and IRS2 levels were highest in the control and HGID + LSFN groups, and high-dose SFN supplementation suppressed IRS1 independently of timing. Exposure to HGID downregulated the expression of PGC-1α and sirtuins. SIRT1 and SIRT3 gene expressions showed a significant increase at both doses, whereas SIRT2 gene expression increased significantly only at 5 mg/kg/day SFN. FASN expression was upregulated in all HGID-fed groups with or without SFN intervention. **Conclusions:** SFN may reverse the adverse effects of HGID in a time- and dose-dependent manner by regulating postprandial insulin, inhibiting gluconeogenesis, and enhancing fatty acid oxidation through the activation of sirtuins and PGC-1α.

## 1. Introduction

Obesity is a global public health problem that is becoming increasingly prevalent due to changing dietary patterns, quality, and eating habits, termed the “nutrition transition” [1]. Previous analyses using various methods indicate that global health expenditures caused by obesity and its complications could rise to 20% [2,3]. Furthermore, the increase in the incidence of obesity in children and adolescents points to the future global health and economic burden caused by chronic diseases associated with obesity [4]. Therefore, the prevention of obesity is critical in the control of obesity-related non-communicable diseases (NCDs), including type 2 diabetes (T2DM), insulin resistance, metabolic syndrome, hyperlipidemia, and non-alcoholic fatty liver disease (NAFLD) [2]. The high consumption of foods with a high glycemic index (GI) and glycemic load and of beverages sweetened with sugar or artificial sweeteners triggers the risk of developing these diseases in the general population, particularly in high-risk groups [5,6]. Hyperglycemia and insulin resistance play a key role in the pathogenesis of these diseases [7]. Blood glucose concentration is continuously regulated by a homeostatic regulatory system, and diet-derived glycemia is one of the primary determinants of glucose metabolism causing the greatest variation during the day. Following the consumption of high-GI foods, there is a rapid increase in blood glucose and insulin responses, and hyperglycemia is followed by many pathophysiological events such as dyslipidemia, protein glycation, insulin resistance, endothelial dysfunction, oxidative stress, and inflammation [8]. Thus, increased carbohydrate intake may cause endocrine dysregulation characterized by hyperinsulinemia, increasing energy storage in adipose tissue and directing carbohydrates from metabolically active tissues (heart, lung, liver, etc.) to adipose tissue, leading to adaptive increases in food intake and decreases in energy expenditure [9]. Therefore, approaches aiming to make changes in the quality and quantity of dietary carbohydrates or to prevent postprandial glycemic fluctuations may have advantages [10].

Phytochemicals, one of the subjects addressed to reverse the adverse effects of a high-glycemic-index diet, are bioactive compounds that can be used in the treatment of various diseases or in alternative medicine practices [11]. The phytochemical sulforaphane (SFN) is an isothiocyanate from the glucosinolates group, found stored as glucoraphanin at high levels in vegetables such as cabbage, cauliflower, and kale and especially in broccoli sprouts [12]. There is strong epidemiological evidence regarding the beneficial health effects of broccoli consumption, and it is known that most of these are mediated by SFN [13]. Phytochemicals can be used in the fight against obesity and associated comorbidities. In vitro studies have shown that SFN can inhibit adipocyte differentiation in the early stages of adipogenesis [14], enhance the browning of white adipocytes, stimulate glucose uptake and oxidative utilization while suppressing the triacylglycerol synthesis pathway, increase lipolysis, upregulate the fatty acid oxidation pathway, and support mitochondrial biogenesis [15]. In animal studies, it has been determined that it reduces body weight gain induced by a high-fat diet (HFD), perirenal and epididymal adipose tissue weights, and epididymal adipocyte size [16], and it increases energy expenditure and the protein expression of Uncoupling Protein 1 (UCP1) in fat depots [17].

The cultivation of nutritional sources of sulforaphane typically involves practices that protect soil health and reduce dependence on synthetic inputs. Since it provides spontaneous defense against natural pests due to its chemical composition, it reduces the need for chemical interventions [18]. On the other hand, significant by-products such as leaves and stems are produced in the production of these plants, and the waste of these parts is a problem in terms of sustainability. These by-products have the potential to be used as functional ingredients due to their dietary fiber and phenolic compound contents to improve the nutritional values of different food products. The valorization of these by-products supports the sustainable use of food resources and encourages an increase in the global food quantity available [19]. Furthermore, the green leaves of these plants are a sustainable source of edible plant-based proteins [20]. For these reasons, the inclusion of SFN in nutrition primarily contributes to the achievement of the goal of ‘Ensure healthy lives and promote well-being for all at all ages’ [Sustainable Development Goal (SDG) 3] by supporting human health. Subsequently, by reducing food waste, promoting resource efficiency, offering socio-economic benefits, and mitigating environmental impact, it contributes to the realization of various Sustainable Development Goals (SDGs) such as ‘Responsible consumption and production’ (SDG 12) and ‘Climate action’ (SDG 13) [21].

Results regarding the effects of SFN supplementation in resolving the adverse effects caused by a diet rich in high-glycemic-index foods, which is a part of the changing dietary pattern and quality that is a significant problem of our age, are limited. In this context, this study aimed to determine, in a mouse model, the effects of different doses and timing of SFN supplementation and associated metabolic parameters/pathways in reducing obesity caused by high-glycemic-index diet feeding and in correcting poor glycemic control and dyslipidemia.

## 2. Materials and Methods

### 2.1. Animals and Experimental Design

**Figure 1:**
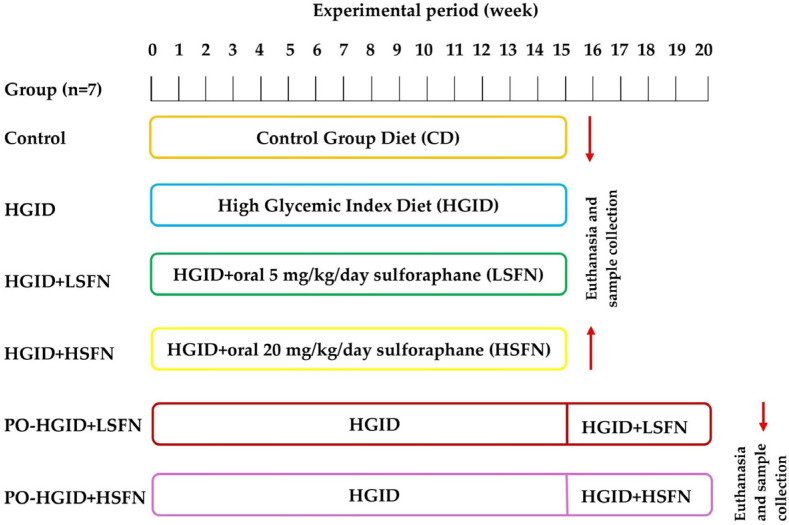
Schematic representation of the experimental design. C57BL/6 mice were administered control diet (CD), high-glycemic-index diet (HGID), HGID + 5 mg/kg/day sulforaphane [HGID+ low-dose SFN (LSFN], and HGID + 20 mg/kg/day SFN [HGID+ high-dose SFN (HSFN)] for 15 weeks and 5 mg/kg/day SFN [post-obesity (PO)-HGID + LSFN] or 20 mg/kg/day SFN (PO-HGID + HSFN) for 5 weeks following 15 weeks of HGID. At the end of the experiment, blood and tissue samples were collected under anesthesia to assess various physiological parameters and changes in gene expression. After sample collection, euthanasia was performed through cervical dislocation. The obtained data were used to evaluate the effects of SFN supplementation on the metabolic dysfunction induced by HGID.

Specific-Pathogen-Free (SPF) C57BL/6 mice (male, eight weeks old, weighing 18–20 g) were purchased from Karadeniz Technical University Animal Laboratory in Turkey, divided into 6 groups by randomization (n = 7), and housed in a controlled environment (22–25 ± 1 °C, 55 ± 10% RH, and 12 h light/dark cycle) with free access to food and water. The experiments were carried out at Erzincan Binali Yıldırım University Experimental Animals Application and Research Center with the approval of the Institutional Animal Ethics Committee (date: 27 July 2023, decision number: 280056). Eight-week-old adult mice were administered a control diet (control), HGID, HGID + oral 5 mg/kg/day SFN [HGID + low-dose SFN (LSFN)], or HGID + 20 mg/kg/day SFN [HGID + high-dose SFN (HSFN)] for 15 weeks and 5 mg/kg/day SFN [post-obesity (PO)-HGID + LSFN] or 20 mg/kg/day SFN (PO-HGID + HSFN) for 5 weeks following 15 weeks of HGID (Figure 1).

The energy, macronutrient, and fiber contents of the HGID used in the experiment and the isocaloric control group feed were kept equal. The only difference between the HGID and the control group feed was the composition of the starch. While the starch in the HGID was a waxy corn starch consisting of 100% amylopectin, it was natural starch (75% amylopectin, 25% amylose) in the control group (Table 1). The macronutrient composition of the feeds used in the experiment was determined similarly to AIN-93M so that the mice were not exposed to significant changes in the macronutrient composition of the diet during the study. SFN was administered to the animals via oral gavage (SFN; 5 mg/kg/day or 20 mg/kg/day). The SFN doses (5 and 20 mg/kg/day) were determined based on commonly used therapeutic dose ranges that have demonstrated biological efficacy without significant signs of toxicity in previously published experimental animal models. Animals were weighed weekly, and feed consumption was recorded twice a week.

At the end of the study, mice were anesthetized with a mixture of ketamine and xylazine (100 mg/kg and 10 mg/kg ip) after 12 h of fasting. Blood was collected via cardiac puncture into tubes with and without anticoagulant. Blood samples were centrifuged at 4000 rpm for 10 min at 4 °C, and the obtained serum was stored at −80 °C for analyses. The liver was dissected and weighed and then stored at −80 °C for further analyses. The experiment was terminated by cervical dislocation under anesthesia.

### 2.2. Glucose Tolerance Test (GTT), Insulin Tolerance Test (ITT)

Glucose tolerance was evaluated using the oral glucose tolerance test (OGTT) method in the control, HGID, HGID + LSFN, and HGID + HSFN groups at the 12th week of diet exposure and in the PO-HGID + LSFN and PO-HGID + HSFN groups at the 19th week. After 6 h of fasting, blood samples were taken from the tail vein of the mice, and fasting blood glucose levels were determined. Then, blood samples were taken from the tail veins at 0, 15, 30, 60, 90, and 120 min after D-(+)-glucose (2 g/kg, Sigma-Aldrich, St. Louis, MO, USA) administration. Glucose concentrations were monitored using an Accu-CHEK Instant Glucometer (Roche, Basel, Switzerland) [22,23]. Three days after OGTT, ITT was performed with intraperitoneal (ip) insulin (0.75 U/kg) injection after the mice were fasted for 6 h. Glucose concentrations were recorded from blood samples taken from tail veins at 0 (before insulin injection), 15, 30, 60, 90, and 120 min using a glucometer. The area under the time–blood glucose curve (AUC) was calculated according to the trapezoidal rule.

### 2.3. HOMA-IR, QUICKI, HOMA-%S, and HOMA-β Scores

Peripheral insulin sensitivity/resistance indices, namely homeostatic model assessment of insulin resistance (HOMA-IR) [24], quantitative insulin sensitivity check index (QUICKI) [25], and homeostasis model assessment S% (HOMA-%S) [26], along with the pancreatic β-cell function index homeostatic model assessment β (HOMA-β) [24], were calculated using the following formulas:

- HOMA-IR = {Fasting serum glucose (mg/dL) × Fasting serum insulin (mIU/L)}/405;
- QUICKI = 1/{log [Fasting blood glucose (mg/dL)] + log [Fasting insulin (mIU/L)]};
- HOMA-%S = {1/HOMA-IR} × 100;
- HOMA-%β = {20 × Fasting serum insulin (mIU/L)}/{Fasting blood glucose (mmol/L) − 3.5}.

### 2.4. Liver to Body Weight Ratio

The liver to body weight ratio was calculated using the equation specified below:

- Liver index = {Liver weight (g)/body weight (g)} × 100.

### 2.5. Biochemical Analyses

HbA1c (HbA1C, Otto Scientific, Ankara, Turkey) levels in whole-blood samples were determined using standard kits; total cholesterol (TC) (Mouse Total Cholesterol ELISA Kit, Bioassay Technology Laboratory, Shanghai, China) and triglyceride (TG) (Mouse Triglyceride ELISA Kit, Bioassay Technology Laboratory, Shanghai, China) levels in plasma samples; and glucose (Glucose Assay Kit, Bioassay Technology Laboratory, Shanghai, China), insulin (Mouse, Insulin ELISA Kit, Bioassay Technology Laboratory, Shanghai, China), and adiponectin (Mouse Adiponectin ELISA Kit, Bioassay Technology Laboratory, Shanghai, China) levels in serum blood samples were determined using ELISA kits. Liver tissue samples were homogenized and mixed with PBS at a 1:9 ratio. Tissue suspensions were subjected to freeze–thaw three times, then centrifuged at 5000 rpm for 10 min, and the supernatant part was separated. The obtained supernatant was transferred to new tubes, and insulin receptor (IR) (Mouse Insulin Receptor ELISA Kit, Bioassay Technology Laboratory, Shanghai, China), insulin receptor substrate 1 protein (IRS1) (Mouse Insulin Receptor Substrate 1 ELISA Kit, Bioassay Technology Laboratory, Shanghai, China), and insulin receptor substrate 2 protein (IRS2) (Mouse Insulin Receptor Substrate 2 ELISA Kit, Bioassay Technology Laboratory, Shanghai, China) levels were determined by the ELISA method.

### 2.6. Quantitative Real-Time PCR Analysis

Total RNA was extracted from the liver using a DiaRex^®^ Total RNA Extraction Kit. (Cat. No: TR-0877-50, Diagen, Ankara, Turkey). The purity and quality of isolated RNA samples were measured with a UV spectrophotometer. Isolated RNA was reverse-transcribed to cDNA using a cDNA kit (Solis Biodyne, Tartu, Estonia). Real-Time PCR was performed in the Bio-Rad Real-Time PCR Detection System using SYBR green chemistry. Primer sequences are given in Table 2. Relative quantification of target gene expression was calculated using the 2^−ΔΔCt^ method. Relative gene expression levels were normalized to β-actin.

### 2.7. Evaluation of Data

The obtained data were analyzed with the SPSS (Statistical Package for Social Sciences) 22.0 statistical software and presented as graphs. Data were presented as mean ± standard deviation (SD), and differences between groups were evaluated using one-way analysis of variance (ANOVA). If the results of the one-way ANOVA test were significant, differences between groups were estimated using the Tukey post hoc test. Repeated-measures ANOVA was used for GTT, ITT, body weight, and feed consumption. Statistical significance was accepted at the *p* < 0.05 level.

## 3. Results

### 3.1. Sulforaphane (SFN) Reduces Diet-Induced Obesity

**Figure 2:**
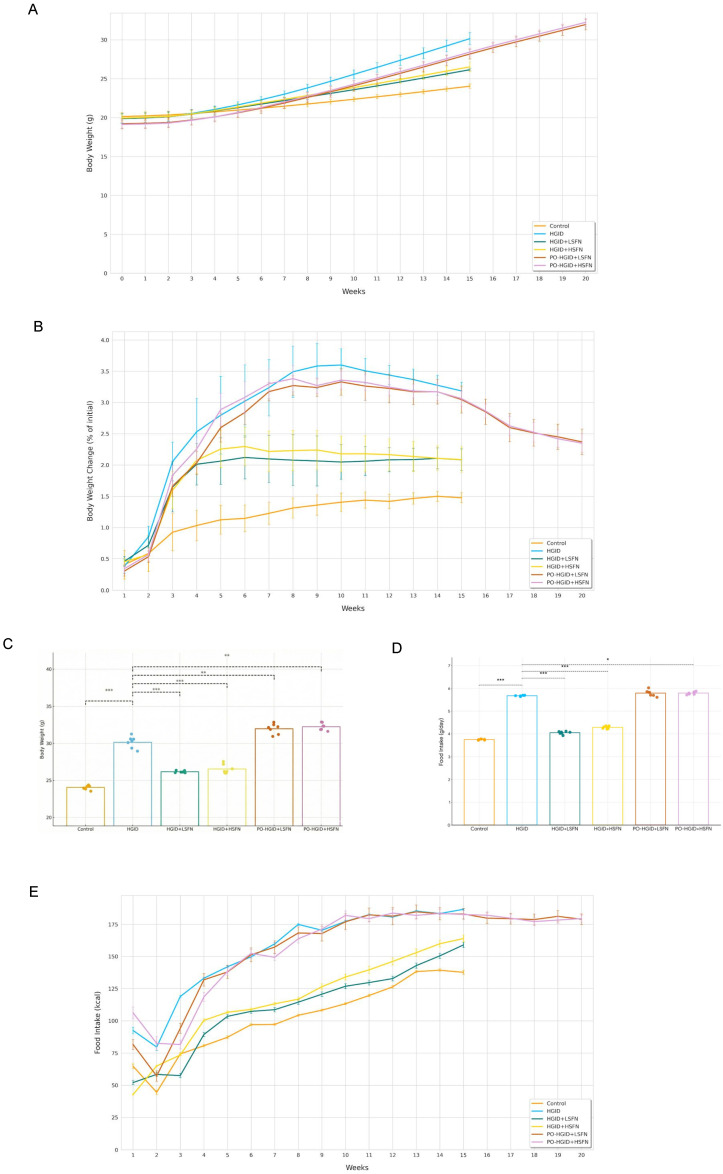

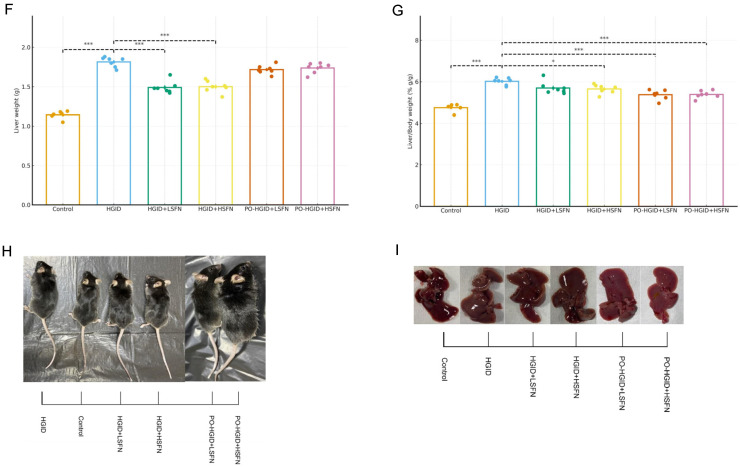
Effect of sulforaphane (SFN) on body weight, energy consumption, and liver in mice fed with high-glycemic-index diet (HGID) and made obese with HGID. (**A**) Body weight measured weekly (g). (**B**) Percentage change in body weight (%). (**C**) Final body weight (g) measured at the end of 15 weeks in control, HGID, HGID + low-dose SFN (LSFN), and HGID + high-dose SFN (HSFN) groups and at the end of 20 weeks in post-obesity (PO)-HGID + LSFN and PO-HGID + HSFN groups. (**D**) Daily mean food intake (g/day). (**E**) Weekly mean energy consumption (kcal). (**F**) Liver tissues of mice were harvested and weighed at the end of the study (g). (**G**) Liver index was calculated using the formula: liver weight (g)/body weight (g) × 100. (**H**) Images of mice. (**I**) Images of mouse livers. Data are presented as mean ± SD. Group differences were assessed using one-way ANOVA followed by the Tukey post hoc test. * *p* < 0.05, ** *p* < 0.01, *** *p* < 0.001 indicate significant difference from the HGID group. Dashed lines are used to show statistical comparisons between the HGID group and others. Each point represents an individual mouse, and diet groups are denoted by color (control: orange; HGID: blue; HGID + LSFN: green; HGID + HSFN: yellow; PO-HGID + LSFN: red; PO-HGID + HSFN: purple).

To evaluate the potential role of SFN on energy metabolism, the effects of SFN were tested by administering it concurrently with HGID and after obesity was induced by HGID. The body weights of mice fed with HGID started to increase significantly from the second week compared to those fed with normoglycemic feed (Figure 2A). In mice administered SFN in addition to HGID, weekly body weight gain was higher compared to the control but lower compared to the HGID group (Figure 2B). At the end of 15 weeks, body weights in the HGID group (30.15 ± 0.8 g) were statistically significantly higher than in the control (24.04 ± 0.3 g), HGID + 5 mg/kg/day SFN (26.15 ± 0.1 g), and HGID + 20 mg/kg/day SFN (26.52 ± 0.6 g) groups (*p* < 0.001) (Figure 2C). In mice made obese with HGID, the increase in body weight gain slowed down following SFN administration (Figure 2B). The decreasing rate of body weight gain was accompanied by a significant reduction in energy consumption (Figure 2E). In the experiment, SFN treatment significantly reduced food consumption and body weight gain when applied concurrently with HGID or after obesity developed with HGID. Liver weight in HGID-fed mice was approximately 37% higher compared to the control group (Figure 2F). Compared to HGID-fed mice, liver weight was approximately 17.7% lower in mice treated with HGID + 5 mg/kg/day SFN and 17.1% lower in those treated with HGID + 20 mg/kg/day SFN (Figure 2F). While the liver weight/body weight index of the mice was lowest in the control group, this was followed by the groups administered SFN in addition to HGID and the HGID group, respectively (Figure 2G) (*p* < 0.05).

### 3.2. Sulforaphane (SFN) Regulates Glucose and Insulin Tolerance

**Figure 3:**
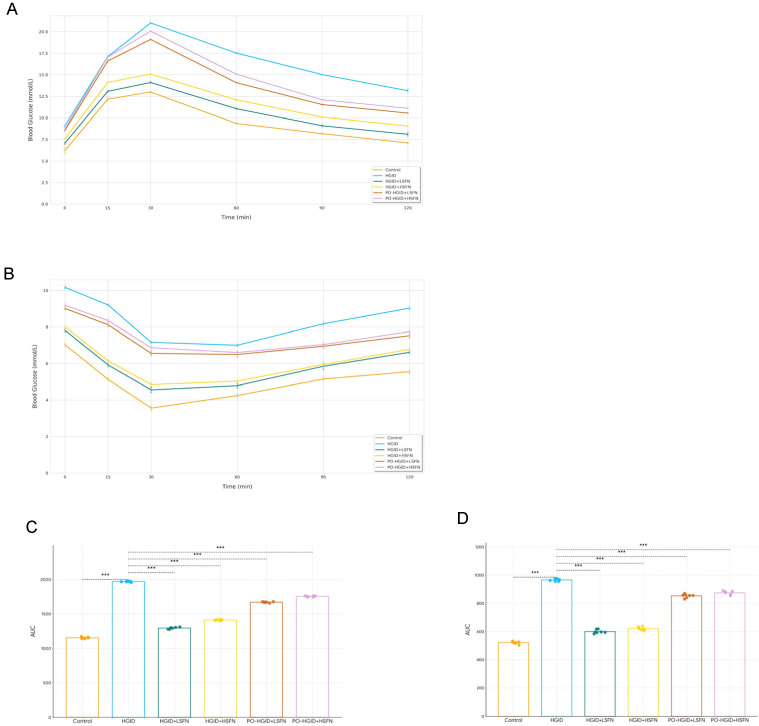
Effect of sulforaphane (SFN) on glucose tolerance and insulin tolerance. (**A**) Glucose tolerance test (GTT). (**B**) Insulin tolerance test (ITT). (**C**) AUC (area under the curve of GTT). (**D**) AUC (area under the curve of ITT). GTT and ITT were performed at the 12th week of dietary exposure in control, high-glycemic-index diet (HGID), HGID + low-dose SFN (LSFN), and HGID + high-dose SFN (HSFN) groups and at the 19th week in post-obesity (PO)-HGID + LSFN and PO-HGID + HSFN groups. Data are presented as mean ± SD. Group differences were assessed using one-way ANOVA followed by the Tukey post hoc test., *** *p* < 0.001 indicate significant difference from the HGID group. Dashed lines are used to show statistical comparisons between the HGID group and others. Each point represents an individual mouse, and diet groups are denoted by color (control: orange; HGID: blue; HGID + LSFN: green; HGID + HSFN: yellow; PO-HGID + LSFN: red; PO-HGID + HSFN: purple).

To understand whether sulforaphane supplementation plays a role in altering insulin resistance, GTT and ITT were performed to evaluate glucose homeostasis and insulin sensitivity in mice. Feeding with a high-glycemic-index diet for 12 weeks raised fasting blood glucose levels, which was indicative of decreased hepatic insulin sensitivity. Mice in the HGID group exhibited impaired glucose clearance compared to the control and were less sensitive to insulin. SFN supplementation in HGID-fed mice showed remarkable improvement in glucose and insulin tolerance (Figure 3A,B). Additionally, only in the HGID and PO-HGID + SFN groups, the decline in blood glucose continued until the 60th minute during insulin tolerance tests (ITT); this result was not observed in the control group and groups receiving SFN in addition to HGID (Figure 3B).

### 3.3. Sulforaphane (SFN) Improves Peripheral Insulin Sensitivity/Resistance and Pancreatic β-Cell Function

**Figure 4:**
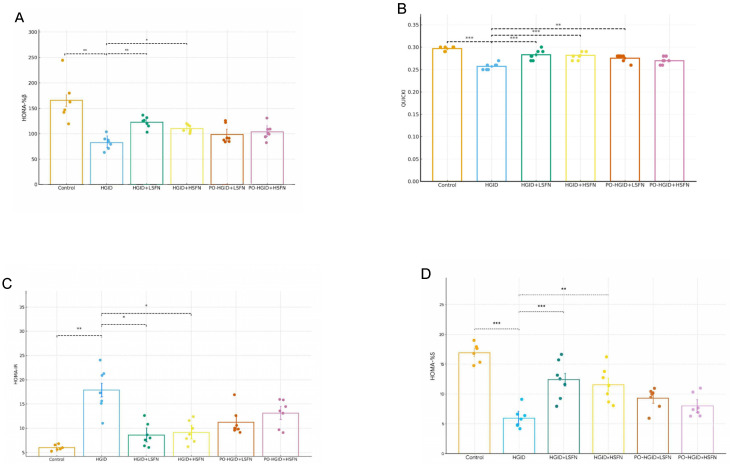
Effect of sulforaphane (SFN) on pancreatic β-cell function and peripheral insulin sensitivity and resistance parameters. (**A**) Homeostatic model assessment of β-cell function percentage (HOMA-%β). (**B**) Quantitative insulin sensitivity check index (QUICKI). (**C**) Homeostatic model assessment of insulin resistance (HOMA-IR). (**D**) Homeostasis model assessment of insulin sensitivity percentage (HOMA-%S). Data are presented as mean ± SD. Group differences were assessed using one-way ANOVA followed by the Tukey post hoc test. * *p* < 0.05, ** *p* < 0.01, *** *p* < 0.001 indicate significant difference from the high-glycemic-index diet (HGID) group. Dashed lines are used to show statistical comparisons between the HGID group and others. Each point represents an individual mouse, and diet groups are denoted by color (control: orange; HGID: blue; HGID + low-dose SFN (LSFN): green; HGID + high-dose SFN (HSFN): yellow; post-obesity (PO)-HGID + LSFN: red; PO-HGID + HSFN: purple).

During the experiment, HGID feeding worsened peripheral insulin sensitivity and resistance indices and the pancreatic β-cell function index. In groups administered low and high doses of SFN in addition to HGID, HOMA-%β, HOMA-%S, and QUICKI scores were statistically significantly higher, and the HOMA-IR score was lower compared to the HGID group (*p* < 0.05) (Figure 4). When 5 mg/kg/day SFN was supplemented to mice made obese with HGID, HOMA-%β and HOMA-%S scores increased and HOMA-IR score decreased, but only the increase in the QUICKI score created a statistically significant difference (Figure 4A–D).

### 3.4. Sulforaphane (SFN) Ameliorates Metabolic Function Worsened by HGID

**Figure 5:**
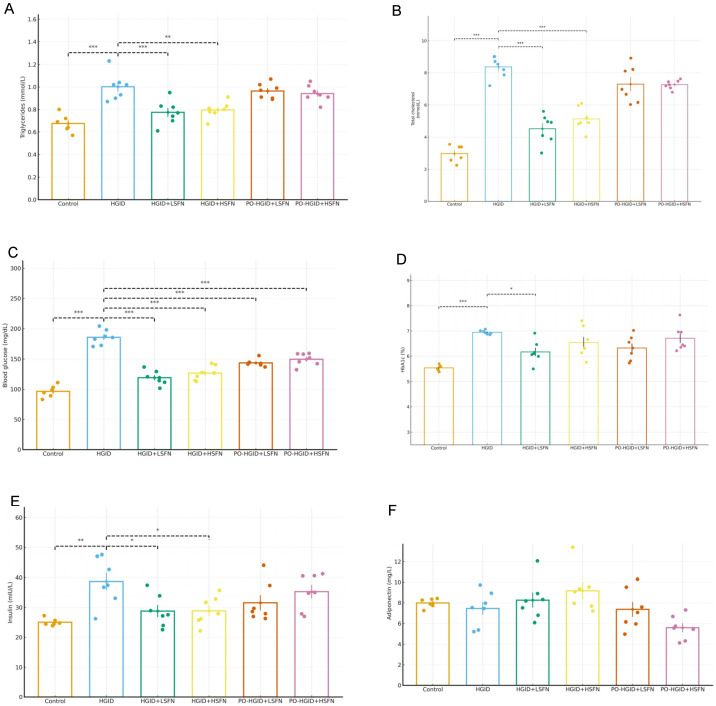
Effect of sulforaphane (SFN) on biochemical blood parameters. (**A**) Triglyceride (mmol/L) determined in plasma. (**B**) Total cholesterol (mmol/L) determined in plasma. (**C**) Serum glucose (mg/dL). (**D**) Hemoglobin A1c (HbA1c) determined in whole blood (%). (**E**) Insulin hormone determined in serum (mIU/L). (**F**) Adiponectin hormone determined in serum (mg/L). Data are presented as mean ± SD. Group differences were assessed using one-way ANOVA followed by the Tukey post hoc test. * *p* < 0.05, ** *p* < 0.01, *** *p* < 0.001 indicate significant difference from the high-glycemic-index diet (HGID) group. Dashed lines are used to show statistical comparisons between the HGID group and others. Each point represents an individual mouse, and diet groups are denoted by color (control: orange; HGID: blue; HGID + low-dose SFN (LSFN): green; HGID + high-dose SFN (HSFN): yellow; post-obesity (PO)-HGID + LSFN: red; PO-HGID + HSFN: purple).

As shown in Figure 5A,B, TC and TG levels were lower in the control group and groups receiving HGID + SFN supplementation compared to mice fed only HGID and mice administered SFN after developing obesity with HGID (*p* < 0.001). Serum glucose was determined to be lowest in the control group mice (96.57 ± 1.0 mg/dL) (Figure 5C). This was followed by HGID + 5 mg/kg/day SFN (119.02 ± 11.6 mg/dL), HGID + 20 mg/kg/day SFN (126.67 ± 11.8 mg/dL), PO-HGID + 5 mg/kg/day SFN (143.63 ± 5.8 mg/dL), PO-HGID + 20 mg/kg/day SFN (149.52 ± 10.1 mg/dL), and mice in the HGID group (185.70 ± 12.4 mg/dL), respectively (Figure 5C). HbA1c percentages of the mice were lowest in the control group (5.54 ± 0.1) and the HGID + 5 mg/kg/day SFN group (6.16 ± 0.4) and were statistically significantly lower in both groups compared to HGID (Figure 5D) (*p* < 0.001). When evaluated in terms of hormone parameters, serum insulin levels were found to be lower in control, HGID + LSFN, and HGID + HSFN mice compared to other groups (Figure 5E). SFN supplementation increased serum adiponectin levels when administered in addition to HGID, but it was not found to be statistically different from the HGID group (Figure 5F).

### 3.5. Sulforaphane (SFN) Restores IRS1/IRS2 Function Deteriorated by HGID

**Figure 6:**
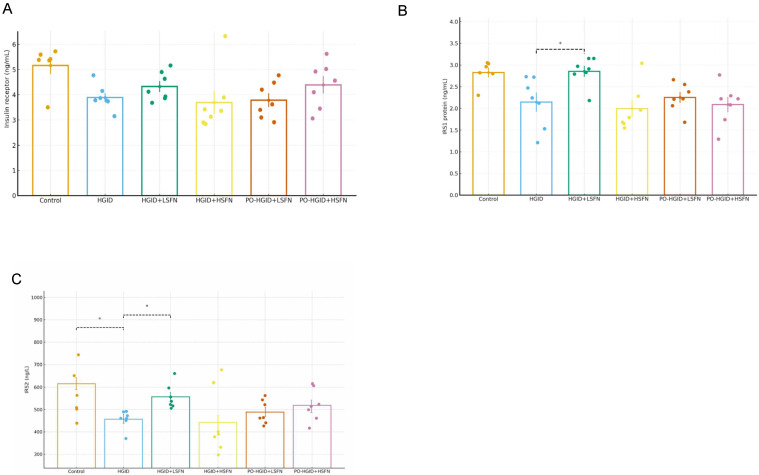
Effect of sulforaphane (SFN) on insulin receptor and insulin receptor substrate proteins. (**A**) Insulin receptor level (ng/mL) determined in liver tissue. (**B**) Insulin receptor substrate 1 protein (IRS1) level (ng/mL). (**C**) Insulin receptor substrate 2 protein (IRS2) level (ng/L). Data are presented as mean ± SD. Group differences were assessed using one-way ANOVA followed by the Tukey post hoc test. * *p* < 0.05, indicate significant difference from the high-glycemic-index diet (HGID) group. Dashed lines are used to show statistical comparisons between the HGID group and others. Each point represents an individual mouse, and diet groups are denoted by color (control: orange; HGID: blue; HGID + low-dose SFN (LSFN): green; HGID + high-dose SFN (HSFN): yellow; post-obesity (PO)-HGID + LSFN: red; PO-HGID + HSFN: purple).

Although SFN provides minor improvements in receptor levels that decline as a result of HGID, this difference is not statistically significant (Figure 6A). IRS1 and IRS2 protein levels, which decreased with HGID, increased with HGID + 5 mg/kg/day SFN administration (Figure 6B,C) (*p* < 0.05). Furthermore, high-dose SFN suppressed the IRS1 protein independently of timing (Figure 6B).

### 3.6. SFN Prevents HGID-Induced Obesity Development by Regulating Gene Expressions of PGC-1α and Sirtuins in Liver Tissue

**Figure 7:**
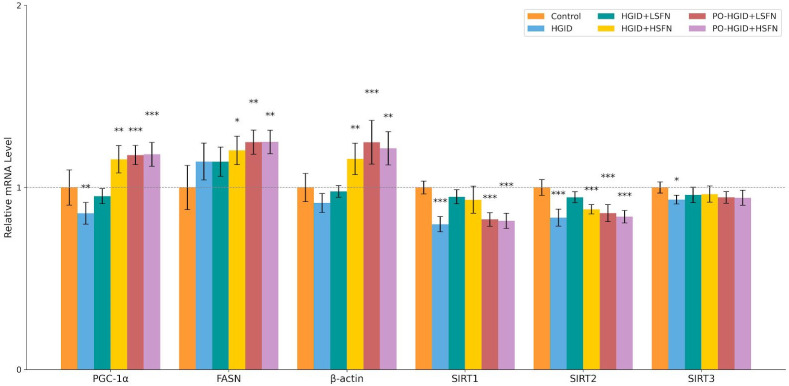
Effect of sulforaphane (SFN) on gene expression of PGC-1α, FASN, SIRT1, SIRT2, and SIRT3 in liver tissue. Gene expressions were analyzed by qPCR. Data are normalized to housekeeping genes and presented as mean ± SD. * *p* < 0.05, ** *p* < 0.01, *** *p* < 0.001 indicate significant difference from the control group. The dashed line represents the control group (set to 1), with other groups adjusted accordingly. (Control: orange; high-glycemic-index diet (HGID): blue; HGID + low-dose SFN (LSFN): green; HGID + high-dose SFN (HSFN): yellow; post-obesity (PO)-HGID + LSFN: red; PO-HGID + HSFN: purple). FASN: FASN, fatty acid synthase gene; PGC-1α: peroxisome proliferator-activated receptor gamma coactivator 1-alpha; SIRT: sirtuin.

mRNA expression levels of PGC-1α, FASN, SIRT1, SIRT2, and SIRT3 were measured using Real-Time PCR. PGC-1α expression in the HGID group was significantly lower compared to the control group. PGC-1α mRNA expression levels in treatment groups (HGID + SFN groups and PO-HGID + SFN groups) were higher compared to the HGID group. Conversely, exposure to HGID upregulated FASN expression in all groups with or without SFN intervention. Briefly, SFN treatment effectively increases PGC-1α expression at the HGID + 5 mg/kg/day dose but does not affect FASN expression. Feeding with HGID downregulated the expression of SIRT1, SIRT2, and SIRT3. Intervention with SFN in PO-HGID + SFN groups did not significantly change SIRT1 mRNA expression levels. SIRT1 and SIRT3 gene expressions, which fell during intervention with HGID, increased in HGID + SFN groups and followed a similar course to the control group. SIRT2 mRNA expression levels increased significantly only in the HGID + 5 mg/kg/day SFN group and were found to be similar to the control group (Figure 7).

## 4. Discussion

### 4.1. Interpretation of Results and Potential Metabolic Pathways

To our knowledge, this is the first study investigating the therapeutic effects of SFN on obesity resulting from feeding with high-glycemic-index foods. To evaluate the potential role of SFN on energy metabolism, obesity development, and insulin resistance, the effects were tested by administering SFN at different doses in addition to HGID and after animals were made obese with HGID.

The carbohydrate composition of the diet may influence the risk of common chronic diseases such as obesity, diabetes, and cardiovascular diseases through changes in postprandial insulin hormone and glucose concentrations [27,28]. In research conducted on experimental animals, high GI diets have been associated with increases in fat mass, insulin resistance [27,29,30], lipogenic gene expression [30], and TG concentration [31] and associated with decreases in lean body mass and adiponectin levels [31]. However, the molecular mechanisms by which high-GI diets exert their effects are not fully known. In summary, continuous and excessive calorie intake from high-glycemic-index foods leads to rapid and frequently recurring hyperglycemia, hyperinsulinemia, and an increase in pro-inflammatory factors, causing insulin resistance [32,33]. Increased serum insulin concentration with a carbohydrate-rich diet promotes *de novo* lipogenesis in the liver. *De novo* lipogenesis is the process where a cell converts excess carbohydrates into fatty acids via acetyl-coenzyme A (acetyl-CoA) [34]. Hyperglycemia developing after a meal with a high glycemic load causes an excessive increase in acetyl-CoA release. The entry of acetyl-CoA into the citric acid cycle produces ATP and free radicals; this chronic overload exacerbates oxidative stress [33]. In the cytoplasm, acetyl-CoA is converted by acetyl-CoA carboxylase (ACC) to malonyl-CoA for the synthesis of fatty acids and subsequently to palmitate by FAS. Palmitate is then used in the synthesis of TG and very-low-density lipoprotein (VLDL). Malonyl-CoA acts as an inhibitor of the carnitine/palmitoyl shuttle system for fatty acid oxidation. High malonyl-CoA levels inhibit carnitine/palmitoyl-transferase 1 (CPT-1) and reduce fatty acid β-oxidation [35]. The key transcription factors regulating lipogenic genes in response to glucose and insulin are sterol regulatory element-binding proteins (SREBP-1 and SREBP-2), liver X receptors, and carbohydrate response element-binding protein (ChREBP) [36]. Glucose induces the dephosphorylation of ChREBP, which activates lipogenic genes [37]. SFN, whose activity against HGID-induced obesity was investigated in this study, has a complex molecular profile that activates multiple cellular pathways both directly and indirectly. For instance, while SFN activates AMP-activated protein kinase (AMPK) [16,38], it induces the dissociation and activation of Nrf2 by interacting with reactive cysteine residues of Kelch-like ECH-associated protein 1 (Keap1) [39,40]. Phosphorylation of AMPK suppresses *de novo* lipogenesis by reducing ACC activity and lowering malonyl-CoA levels, leads to increased mitochondrial fatty acid oxidation [41,42], and lowers cholesterol levels by inhibiting HMG-CoA reductase, the rate-limiting enzyme in cholesterol biosynthesis [43]. Moreover, AMPK downregulates the expression of genes related to lipogenesis by directly phosphorylating SREBP-1c [44] and can stimulate SIRT1 activity by increasing NAD+ levels [45]. Through phosphorylation by AMPK and deacetylation by SIRT1, PGC-1α is activated, thereby promoting mitochondrial biogenesis and oxidative metabolism [45]. In the cytoplasm, SIRT2 can suppress cytosolic acetyl-CoA production and, indirectly, de novo lipogenesis. It can also deacetylate PGC-1α, thereby promoting the transcription of gluconeogenic enzyme genes. Decreased SIRT2 expression leads to an increase in FASN and other lipogenesis-related genes, promoting lipid accumulation [46]. In the mitochondria, SIRT3 enhances fatty acid oxidation, modulates the effects of PGC-1α, and contributes to the improvement of metabolic homeostasis and reduction in lipid accumulation by reducing the production of reactive oxygen species (ROS). Conversely, suppression of PGC-1α reduces SIRT3 expression [47]. In general, sirtuins, primarily SIRT1, strengthen IRS-1/2 signaling by directly deacetylating IRS2, suppressing inflammation via NF-κB inhibition, and indirectly restricting serine phosphorylation of IRS1 [48]. These mechanisms act as complementary mechanisms breaking the insulin resistance cycle.

In this study, it was observed that feeding with HGID for 15 weeks led to hyperglycemia and hyperinsulinemia. This resulted in the suppression of the insulin receptor (IR) and insulin receptor substrates IRS1 and IRS2 in the liver. Real-Time PCR results suggest that HGID feeding increased lipogenesis and cholesterologenesis through the upregulation of FASN expression, which catalyzes fatty acid synthesis in the liver. These findings are supported by plasma TC and TG levels, confirming that cholesterol and triglyceride biosynthesis significantly increased in the liver (Figure 5A,B). This process is followed by an increase in malonyl-CoA levels, suppression of mitochondrial fatty acid oxidation, and an increase in lipid accumulation and oxidative stress [35]. However, this anabolic phenotype in fat metabolism is not supported by the upregulation of PGC-1α deacetylated by SIRT1, which stimulates hepatic gluconeogenesis and β-oxidation of fatty acids. While PGC-1α expression, which fell with HGID, increased and approached the control group with 5 mg/kg/day SFN supplementation in addition to HGID; 20 mg/kg/day SFN in addition to HGID caused overexpression of PGC-1α. Furthermore, high-dose SFN application also negatively affected the gene expression profile of FASN. SIRT1 and SIRT3 gene expressions, which decreased during intervention with HGID, increased with low-dose or high-dose SFN application and followed a course similar to the control group, but SIRT2 gene expression showed a significant increase only at low-dose SFN. Despite the significant increase in FAS activity in groups administered SFN in addition to HGID, there was a significant decrease in de novo fatty acid synthesis and TG accumulation in the liver. This metabolic contradiction can be explained by SFN-induced AMPK activation leading to the downregulation of cytoplasmic malonyl-CoA levels [41,42]. Reduced malonyl-CoA supports the utilization rather than storage of fatty acids by facilitating the entry of fatty acids into the mitochondria and increasing their β-oxidation. Thus, while a carbohydrate-rich diet induces FAS transcription [35], overall lipid accumulation may be suppressed due to increased fatty acid oxidation. It is possible that SFN converts energy metabolism to a catabolic phenotype by activating sirtuins and PGC-1α, stimulating hepatic gluconeogenesis and β-oxidation of fatty acids. This is thought to form the basis of SFN’s potential against the adverse metabolic effects of high-glycemic-index nutrition. When SFN was administered in addition to the HGID diet, it provided a reduction in body weight, liver weight, and food intake compared to HGID at both doses (*p* < 0.001) and regulated the lipid profile (*p* < 0.01). This supports the idea that SFN can promote weight loss by increasing cellular energy expenditure and fatty acid oxidation. Additionally, sirtuins stimulated by SFN may improve glucose tolerance by regulating insulin secretion in postprandial satiety and suppressing hepatic glucose production. Increased IRS levels in the liver with 5 mg/kg/day SFN administration (Figure 6A,B) predicted decreased gluconeogenesis and increased lipolysis. This was accompanied by suppressed insulin secretion and significant decreases in fasting blood glucose and HbA1c, the indicator of long-term blood glucose control.

In the current study, in models developed to provide evidence on whether SFN can reverse this condition in mice with established diet-induced obesity, insulin resistance, and glucose intolerance, while there was overexpression in the PGC-1α and FASN in PO-HGID + SFN groups, no significant increase was observed in SIRT1 and SIRT2 expression or IRS1 and IRS2. In fact, high-dose SFN suppressed IRS1 just as in the 15-week SFN intervention. Additionally, no significant improvement was observed in insulin levels and lipid profiles at either dose. SFN administered while exposure to HGID continued after diet-induced obesity significantly reduced food consumption and body weight gain and improved hyperglycemia but did not alter hyperinsulinemia.

Previous studies have mentioned that exposure to high-GI diets increases fat mass, TG concentration, and markers of insulin resistance [30,31]. In this study, mice exposed to HGID throughout the experiment exhibited an increase in body weight and liver weight, deterioration in lipid profile, decreased liver insulin sensitivity and, consequently, the development of insulin resistance; these findings are consistent with the literature [30,31]. The continuous rate of increase in body weight observed in HGID-fed mice, especially in the first 10 weeks of the experiment, was accompanied by an increase in food consumption from the 2nd week of the experiment. SFN application in addition to HGID for 15 weeks suppressed body weight gain throughout the experiment. It is noteworthy that despite the increase in energy consumption, especially from the 6th week, the rate of body weight gain slowed down. This observation indicates that SFN may need a certain period to modulate the adverse metabolic effects of HGID feeding; according to this study, this period may be 6 weeks, which deserves further investigation. A similar situation was observed in PO-HGID + SFN groups; the increase in body weight gain slowed down following SFN application. The results coincide with data showing that SFN supplementation significantly reduces body weight increase in diet-induced obesity studies [16,49,50,51]. In this study, SFN provided significant improvement in hepatic steatosis. While feeding with a high-glycemic-index diet caused a significant increase in liver and liver/body weight indices in mice, low- and high-dose SFN supplementation in addition to HGID reversed this situation (*p* < 0.001). In a study investigating the role of SFN in NAFLD, findings consistent with this research revealed that following a 12-week HFD intervention, the group receiving SFN supplementation in addition to the HFD for 6 weeks exhibited lower liver/body weight indices compared to mice fed only the HFD (*p* < 0.05) [52].

While the lipid profile that rose (deteriorated) as a result of HGID feeding did not show significant improvement in groups administered SFN after obesity developed, it decreased significantly in groups receiving SFN supplementation together with HGID (*p* < 0.001). Supporting these findings, low-dose (5 mg/kg) SFN 3 days a week in addition to 10 weeks of HFD significantly reduced only plasma TC and TG, while medium-dose (10 mg/kg) SFN and high-dose (20 mg/kg) SFN reduced liver TC and TG in addition to plasma TC and TG [53]. Another study reported that 20 mg/kg SFN supplementation 3 days a week suppressed the increase in liver weight, serum TC, TG, low-density lipoprotein (LDL), liver TG, and lipid droplets developed by exposure to 10 weeks of HFD [54]. On the other hand, there are also study results in the literature reporting that SFN supplementation was not found to be effective on obesity developed by diet, as in this study [55]. For example, it was reported that 1 mg/kg oral SFN treatment for 16 weeks was not effective on serum lipids and hepatic and renal markers, except for liver triacylglycerols, in preventing or ameliorating metabolic disorders developing as a result of feeding with a high-sucrose diet for 24 weeks [55]. Although the reasons underlying the inconsistency between the results here and previous studies are currently unclear, it is thought that it may stem from differences in the timing of SFN intervention or SFN doses.

GTT and ITT results showed that HGID raised fasting blood glucose levels, impaired glucose clearance, and consequently decreased liver insulin sensitivity. Another remarkable finding is that the decrease in blood glucose continued until the 60th minute during ITT only in HGID and PO-HGID + SFN groups (Figure 3B). This situation stems from the impaired glucose counter-regulation caused by a long-term high-glycemic-index diet and, possibly, from the loss of metabolic flexibility, i.e., the impaired substrate shift that delays the transition to both carbohydrate and fat oxidation in the postprandial period [30]. SFN administration seems to have partially restored this dynamic regulation of glucose homeostasis (metabolic flexibility). This result was supported by the increase in HOMA-%β, HOMA-%S, and QUICKI scores and the decrease in HOMA-IR score in groups administered HGID + SFN (*p* < 0.05). When 5 mg/kg/day SFN was supplemented to mice made obese with HGID, HOMA-%β and HOMA-%S scores increased, HOMA-IR score decreased, but only the increase in QUICKI score was reflected in statistical difference. In approximately 9 weeks of exposure to a high-fructose diet, 0.5 mg/kg/day SFN administration in the 6th week provided significant improvement in GTT’s area under the curve (AUC) and HOMA-IR, while it did not affect ITT’s area under the curve [56]. In another study where the dose of SFN was ≤0.5 mg/kg/day, subcutaneous injection of 0.5 mg/kg SFN 5 days a week for 4 months after exposure to HFD for 3 months did not affect the area under the curve of ITT [57]. In this study, while SFN reduced serum glucose in all groups, it was able to suppress insulin only in groups administered HGID + SFN. In glycosylated hemoglobin measurement performed to confirm the effect of SFN on fasting blood glucose, while there was no significant change in HbA1c in the group administered high-dose SFN, HbA1c level decreased significantly in low-dose SFN administration (Figure 5D). These results are consistent with the literature [17,58]. It was shown that in young male mice fed HFD supplemented with 0.5 g/kg SFN for 20 weeks, SFN improved insulin sensitivity by 23.66% while not altering glucose tolerance. In the same study, providing 0.25 g/kg SFN with a standard diet for 7 weeks in 8-month-old diet-induced obese middle-aged male mice provided an approximately 40% reduction in fasting plasma insulin level compared to the HFD group [51]. Besides preclinical studies, clinical trials have found that SFN reduced glycosylated hemoglobin and fasting blood glucose in T2DM patients [59].

Preclinical studies argue that SFN supplementation plays a role in preventing obesity-related phenotypes by increasing adiponectin levels along with various metabolic pathways [60]. For example, in a study where obesity was induced with HFD, it was shown that 0.1% SFN (1 g sulforaphane/kg diet) could contribute positively to metabolic functions by increasing adiponectin expression [16]. It is known from clinical studies that the high-glycemic-index content of the diet is a strong negative determinant of adiponectin concentration [61,62,63], and chronic inflammation and oxidative stress triggered by high carbohydrate consumption can suppress adiponectin gene expression [64]. In this study, SFN supplementation increased serum adiponectin levels in 20 mg/kg/day SFN rather than 5 mg/kg/day SFN when administered in addition to HGID. However, this difference was not reflected in statistical significance, likely because even the high dose in this study was not sufficient to suppress the suppressive effects of long-term HGID feeding on adiponectin.

In this study, we investigated C57BL/6 mice, which are commonly used in metabolic studies due to their susceptibility to developing diet-induced obesity and hyperglycemia [30], and we fed them either CD or HGID throughout the experiment. While feed consumption decreased in the 2nd week of the experiment during the habituation process to the feed in groups where normoglycemic-index feed (CD) and high-glycemic-index feed (HGID) were administered, food intake did not decrease in the two groups administered SFN treatment. This supports the absence of SFN-induced taste aversion in mice. These findings are consistent with reports from preclinical studies [50,51] and clinical trials [65].

In studies conducted to evaluate the efficacy of SFN on various pharmacological targets, it appears that dose ranges in intraperitoneal and oral administrations are close to each other. The median effective SFN dose with oral administration was determined as 175 µmol/kg body weight and 113 µmol/kg for intraperitoneal administration [13]. The LD50 value of SFN in mice was estimated as 213 mg/kg/ip (1203 µmol/kg) and the TD50 value as 192 mg/kg/ip, and this value is approximately 10 times higher than the median dose reported for efficacy results in mice [66]. In this study, we used the 5 mg/kg SFN dose as it falls within the lower range of previously published studies [50]; the 20 mg/kg SFN dose to reverse the adverse effects of HGID as 20–40 mg/kg SFN is reported as a common and effective treatment dose in mouse models [51,67]. Most doses in animal studies exceed the highest SFN doses used in humans (approximately 10-fold) even after allometric scaling correction between rodents and humans is made. This situation may stem from the higher clearance rate per body weight in rodents compared to humans [13]. Therefore, we acted reasonably when determining the high-SFN dose and mimicked the most appropriate route (oral) for administration to humans in clinical studies [13]. On the other hand, previous studies have shown that a minimum experimental period of 12–13 weeks is needed to observe increased body weight/fat and metabolic changes with HGID in C57BL/6 mice, and a period of at least 16 weeks is needed to develop an obesity-associated phenotype [27,30]. Since it is known that early and longer-term exposure to HGID significantly increases the effect of HGI diets [68], considering the developmental processes (mature) of the mice [69], a high-glycemic-index diet was administered for 15 weeks to 8-week-old adult mice to induce diet-induced obesity. Taking into account the periods reported to be effective in the literature for oral sulforaphane administration, a 5-week SFN treatment protocol was applied after 15 weeks of diet [16,67,70]. Ultimately, although our doses for dose–response analysis are limited, 5 mg/kg/day SFN appears sufficient to reduce weight gain, insulin resistance, and dyslipidemia caused by HGID.

### 4.2. Strengths, Limitations, and Future Directions

This study has several notable strengths. This study is strengthened by its experimental design, in which SFN was administered at multiple doses both during exposure to the HGID and after the development of HGID-induced obesity, allowing for a comprehensive evaluation of its effects on energy metabolism, obesity progression, and insulin resistance. Another strength of this study is its focus on the specific components of the HGID. To be able to separate the effects of the HGI diet pattern, one of the long-standing criticisms regarding GI, from individual components it contains, especially dietary fiber [10,68], we were able to evaluate the glycemic index interaction by keeping the energy, macronutrient, and fiber contents of the feeds equal and the starch composition different in the current study.

Nevertheless, several limitations should be acknowledged. The potential adverse effects of the 5 mg/kg/day and 20 mg/kg/day doses of sulforaphane in this study were evaluated in terms of clinical signs. However, systemic adverse effects, particularly those affecting the brain, cardiovascular system, or other organs, were not assessed. Another limitation is that the relatively short duration of the SFN intervention (five weeks following the development of obesity induced by a HGID) may have limited the ability to fully capture all potential changes in the measured variables. It may be beneficial to observe for a longer period in future studies to provide evidence that SFN reverses HGID-induced obesity. Furthermore, the ideal dose of SFN has not yet been determined. Dose and bioavailability are considered important parameters that need to be clarified for SFN to be considered as an anti-obesity agent. Numerous clinical studies investigating the potential beneficial effects of SFN in a wide range of diseases will ultimately reveal whether the results reported here can be translated to human health.

## 5. Conclusions

The results of this study shed light on the mechanistic basis of the attenuating effects of sulforaphane on obesity and hyperglycemia associated with a high-glycemic-index diet and contribute to the understanding of relevant metabolic pathways in a mouse model. The obtained results indicate that SFN may provide potential benefits both as a protective agent in the obesity development process and as a therapeutic approach after obesity has developed. While SFN suppresses obesity development by combating increased energy consumption, body weight, deteriorated lipid profile, and decreased insulin sensitivity upon exposure to HGID, it supports obesity treatment with its aspects of reducing food consumption and body weight gain and improving glycemic control. SFN may reverse the adverse effects of HGID in a time- and dose-dependent manner by regulating postprandial insulin, restoring IRS1/IRS2 function, inhibiting gluconeogenesis through the coordinated activation of signaling between sirtuins and PGC-1α, and shifting liver metabolism from lipid synthesis toward mitochondrial oxidation. In conclusion, supporting the usability of SFN in the prevention and management of metabolic diseases with preclinical and clinical studies may contribute to reducing the chronic disease burden and associated health expenditures in terms of public health.

## Figures and Tables

**Table 1:** The ingredients and nutritional composition of the experimental diets.

	HGID	CD
Nutrient (g/kg)		
Casein	220	220
L-Cystine DL methionine	3	3
Corn Starch (100% amylopectin)	540	
Corn Starch (75% amylopectin, 25% amylose)		540
Sucrose	82	82
Cellulose	48	48
Corn Oil	52	52
Mineral Mix	43	43
Vitamin Mix	10	10
Choline Bitartrate	2	2
**Contribution of total calories from each macronutrient**		
Protein (energy%)	22.9	22.9
Carbohydrate (energy%)	65.1	65.1
Fat (energy%)	12	12
Total energy	3888 kcal (16.3 kj)/kg	3888 kcal (16.3 kj)/kg

CD: control diet, HGID: high-glycemic-index diet; kcal: kilocalories; kg: kilogram; kj: kilojoule.

**Table 2:** Primer sequences used for qPCR analysis of specific genes.

Gene Name	Forward Primer	Reverse Primer
PGC-1α	CAGACCTAGATACCAACT	CTTCCTTCAGTAAACTATCA
FASN	TCGGTGTATCCTGCTGTC	GGCTTGTCCTGCTCTAAC
SIRT1	AGCTCCTTGGAGACTGCGAT	ATGAAGAGGTGTTGGTGGCA
SIRT2	GCCTGGGTTCCCAAAAGGAG	GAGCGGAAGTCAGGGATACC
SIRT3	ATCCCGGACTTCAGATCCCC	CAACATGAAAAAGGGCTTGGG

FASN: FASN, fatty acid synthase gene; PGC-1α: peroxisome proliferator-activated receptor gamma coactivator 1-alpha; SIRT: sirtuin.

## Data Availability

Dataset available on request from the authors.

## Abbreviations

The following abbreviations are used in this manuscript: AMPK, AMP-activated protein kinase; Acetyl-CoA, acetyl-coenzyme A; ACC, acetyl-CoA carboxylase; AUC, area under the curve; ChREBP, carbohydrate response element-binding protein; CPT-1, carnitine/palmitoyl-transferase 1; FASN, fatty acid synthase gene; GI, glycemic index; GTT, glucose tolerance test; HGID, high-glycemic-index diet; HFD, high-fat diet; HOMA-%β, homeostatic model assessment β%; HOMA-%S, homeostasis model assessment S%; HOMA-IR, homeostatic model assessment of insulin resistance; IR, insulin receptor; IRS1, insulin receptor substrate 1 protein; IRS2, insulin receptor substrate 2 protein; ITT, insulin tolerance test; Keap1, Kelch-like ECH-associated protein 1; LDL, low-density lipoprotein; NAFLD, non-alcoholic fatty liver disease; Nrf2, nuclear factor erythroid 2-related factor 2; PGC-1α, peroxisome proliferator-activated receptor gamma coactivator 1-alpha; QUICKI, quantitative insulin sensitivity check index; SDGs, sustainable development goals; SFN, sulforaphane; SIRT, sirtuin; SREBP, sterol regulatory element-binding proteins; T2DM, type 2 diabetes; TC, total cholesterol; TG, triglyceride; UCP1, uncoupling protein 1; VLDL, very-low-density lipoprotein.
